# Disruption of *TcGNBP3* via RNA interference compromises antibacterial immunity in *Tribolium castaneum*

**DOI:** 10.1080/21505594.2025.2589599

**Published:** 2025-11-14

**Authors:** Jingxiu Bi, Qing Li, Pingxiang Liu, Jingjing Li, Rui Gao, Tong Zhao, Xuexia Yuan, Haining Hao, Yutao Wang, Bin Li

**Affiliations:** a Laboratory of Quality and Safety Risk Assessment for Agro-Products of the Ministry of Agriculture (Jinan), Institute of Quality Standard and Testing Technology for Agro-Products, Shandong Academy of Agricultural Sciences, Jinan, China; b State Key Laboratory of Rice Biology & Breeding, and Zhejiang Provincial Key Laboratory of Crop Germplasm Innovation and Utilization, The Advanced Seed Institute, Zhejiang University, Hangzhou, China; c State Key Laboratory of Nutrient Use and Management, Institute of Quality Standard and Testing Technology for Agro-Products, Shandong Academy of Agricultural Sciences, Jinan, China; d Jiangsu Key Laboratory for Biodiversity and Biotechnology, College of Life Sciences, Nanjing Normal University, Nanjing, China

**Keywords:** GNBP3, *Tribolium castaneum*, innate immunity, RNAi, pest control

## Abstract

Gram-negative bacteria binding proteins (GNBPs) serve as essential pattern recognition receptors in insect innate immunity, detecting pathogen-associated molecular patterns to activate downstream immune responses. This molecular recognition mechanism presents a promising target for pest control strategies. However, the immunological functions of *GNBP* family members in *Tribolium castaneum* remain poorly characterized, particularly for those with typical structural features. In this study, we identified and characterized a novel *GNBP3* (designated *TcGNBP3*) from the *T. castaneum* cDNA library. Structural analysis revealed that TcGNBP3 exhibits the typical domain architecture characteristic of the *GNBP* family, comprising an N-terminal carbohydrate-binding module 39 (CBM39) domain and a C-terminal glycoside hydrolase family 16 (GH16) domain. Spatiotemporal expression profiling demonstrated peak *TcGNBP3* transcript accumulation during the early pupal and late adult developmental stages, with predominant localization in immune-related tissues including the fat body and hemolymph. Bacterial challenges (*Escherichia coli* or *Staphylococcus aureus*) induced significant upregulation of *TcGNBP3* expression from 6 to 72 h. Molecular docking and ELISA analyses demonstrated TcGNBP3’s binding affinity for lipopolysaccharide, peptidoglycan, and β-1,3-glucan, while functional assays confirmed its ability to bind and agglutinate five tested bacterial strains. RNAi-mediated silencing of *TcGNBP3* severely compromised the beetles’ immune response, suppressing immune-related genes (including transcription factors and antimicrobial peptides), disrupting prophenoloxidase cascade activation, and significantly reducing survival rates upon bacterial infection. These results identify *TcGNBP3* as a key immune regulator in *T. castaneum*, supporting its development as an RNAi-based pest control target.

## GRAPHICAL ABSTRACT

**Figure:**
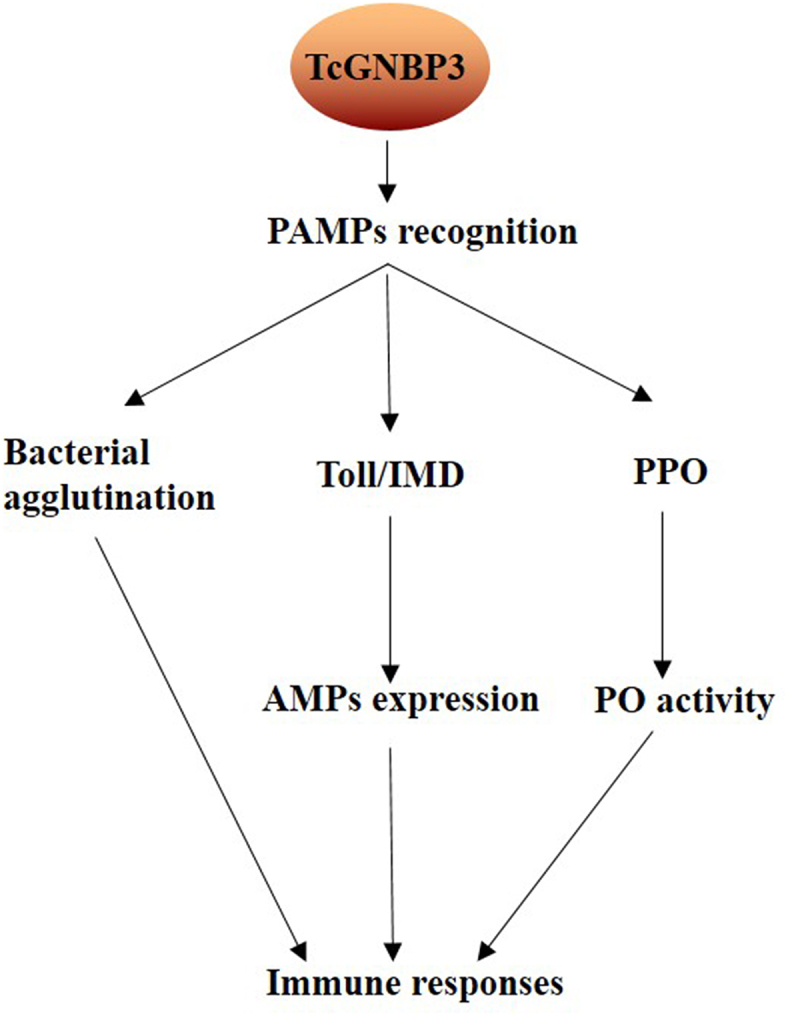

## Introduction

Insects have evolved a sophisticated innate immune system as their primary defense against microbial pathogens, compensating for their lack of vertebrate-style adaptive immunity [1–4]. This sophisticated immune mechanism depends on pattern recognition receptors (PRRs), which identify conserved microbial signatures known as pathogen-associated molecular patterns (PAMPs). These include bacterial components such as peptidoglycan (PGN) and lipopolysaccharide (LPS), as well as fungal molecules like β-1,3-glucans (Glu) [5–8]. Among these PRRs, Gram-negative bacteria binding proteins (GNBPs), also known as β-1,3-glucan recognition proteins (βGRPs), have emerged as crucial first-line defenders in insect immunity [9–11].

Structurally, GNBPs/βGRPs exhibit a conserved domain architecture that reflects their dual functionality in pathogen recognition and immune activation. The N-terminal region usually includes a carbohydrate-binding module 39 (CBM39), which mediates direct binding to different PAMPs, whereas the C-terminal region contains a glycoside hydrolase 16 domain (GH16) responsible for downstream immune signaling [12]. This structural arrangement allows GNBPs to regulate critical immune responses, such as the Toll pathway (activated by fungal and gram-positive bacterial infections), the IMD pathway (targeting gram-negative bacteria), and the prophenoloxidase (proPO) cascade (which drives melanization and pathogen elimination) [6,13,14]. For example, in *Drosophila melanogaster*, upon recognition of fungal cell wall components (β-glucans) by GNBP3, this triggers a cascade that sequentially activates the processing of the Toll Ligand Spätzle, ultimately leading to the expression of antimicrobial peptides (AMPs) to defense fungi [15–17]. Similarly, in the coleopteran insect *Tenebrio molitor*, TmGNBP3 recognizes fungal cell wall component β-1,3-glucan and contributes to antifungal defense through its involvement in the proPO system and induction of downstream antimicrobial peptides [18]. These examples demonstrate the functional diversity of GNBPs in insects, underscoring their critical role in innate immunity against pathogenic challenges. This evolutionary adaptation also highlights their potential as targets for novel pest management strategies.

The growing global crisis of insecticide resistance [19], coupled with increasing environmental concerns [20], has spurred intense interest in RNA interference (RNAi) as a next-generation pest control technology [21,22]. RNAi’s ability to silence essential genes through double-stranded RNA (dsRNA) with high specificity provides unprecedented opportunities for targeted pest management while minimizing ecological impact [23,24]. Recent advances in dsRNA production and delivery methods, including plant-incorporated protectants, nanoparticle formulations, and direct bacterial or fungal delivery systems, have significantly enhanced the practical feasibility of RNAi-based pest control [25–29]. Within this context, GNBPs have emerged as particularly attractive RNAi targets due to their pivotal position in insect immune networks.

The disruption of GNBP function creates a critical vulnerability in pests. While not fatal itself, this impairment weakens their immune defenses, making them more prone to natural infections and reducing their survival capacity [30,31]. This approach targets key physiological systems to selectively compromise pest resilience. Compelling evidence demonstrates the effectiveness of GNBP targeting insect. Following *Beauveria bassiana* infection, *T. molitor* treated with dsTmGNBP3 exhibited increased mortality compared to controls [18]. In *Reticulitermes flavipes*, *GNBP2* suppression led to 2-fold higher mortality upon local strains of *Metarhizium anisopliae* infection [30]. Similarly, RNAi-mediated knockdown of *Tenebrio molitor* GNBP3 significantly compromised host immune defenses against *B. bassiana* JEF-007 infection, leading to reduced survival rates [18]. These consistent findings across diverse species highlight the potential of GNBP targeting for RNAi-based pest control strategies, particularly against coleopteran pests that demonstrate exceptional RNAi sensitivity.

As a model coleopteran storage pest, *Tribolium castaneum* (red flour beetle) causes extensive damage to stored agricultural products, including milled grains, flour, and wheat bran, leading to significant economic losses worldwide. Moreover, the most substantial economic consequences stem from product contamination, which generates negative consumer feedback that damages brand reputation, while also increasing costs associated with product returns and subsequent remediation measures [32,33]. Beyond its role as a destructive storage pest, *T. castaneum* serves as an exceptional model for RNAi research, owing to its strong systemic RNAi response upon dsRNA administration across all life stages. Recent studies have leveraged this species for high-throughput RNAi screening, including investigations into RNAi phenotypes, RNAi-related genes, genome-wide transcriptional responses (RNAiSeq), and the identification of potential pest control targets [32]. Currently, by using RNAi technology of *T. castaneum*, multiple immune-related genes have been identified as potential targets, such as Latrophilin [34], and Lectins [35,36]. Three GNBP family members (*TcGNBP1*, *TcGNBP2*, and *TcGNBP3*) are encoded in the *T. castaneum* genome. However, the immunological functions of *TcGNBP3* remain uncharacterized, leaving its potential innate immune regulatory roles and susceptibility to RNAi-mediated targeting unexplored.

In the present work, we examined the microbial response characteristics of *TcGNBP3* isolated from *T. castaneum*. We then investigated its functional roles in bacterial recognition and agglutination. Using RNAi, we systematically analyzed *TcGNBP3*’s regulatory mechanisms in immune pathways, including AMP gene expression, proPO cascade activation, and host survival rates following bacterial challenge. Our results demonstrate that *TcGNBP3* likely serves as a PRR in innate immunity and represents a potential RNAi target for pest control strategies.

## Materials and methods

### Insect

The Georgia-1 strain of *T. castaneum* obtained from the Bin Li Laboratory of Nanjing Normal University, maintained on whole wheat flour supplemented with 5% brewer’s yeast [37], was used throughout this study. The relative humidity is 40%, the temperature is 30°C, and the photoperiod is 14/10 (bright/dark).

### Cloning and sequencing of TcGNBP3

Three 15-day-old (fifth-instar) *T. castaneum* larvae were pooled as one sample and homogenized in RNAiso™ Plus (TaKaRa, Japan). Total RNA was extracted according to the manufacturer’s instructions. The concentration of the isolated RNA was measured using a NanoDrop spectrophotometer (Thermo Scientific, USA). RNA samples were only used if they met the following quality criteria: A260/A280 ratio > 1.8 and an A260/A230 ratio > 2.0. RNA integrity was further verified by 1% agarose gel electrophoresis. Subsequently, 1 μg of total RNA was reverse-transcribed into cDNA using Reverse Transcriptase M-MLV (TaKaRa, Japan). Based on the *TcGNBP3* sequence from NCBI, gene-specific primers (*TcGNBP3*-F/R, Table 1) were designed using Oligo 6 and employed for PCR amplification. The amplified *TcGNBP3* sequence was re-sequenced to confirm the authenticity.

**Table 1.** Sequences of primers used in this study.

Name	Sequence(5’-3’)	Remarks
*TcGNBP3*-F	ATGAAAGAATTCTCCTTTGTAGT	Clone of *TcGNBP3* ORF
*TcGNBP3*-R	TCAAAGGGCCCATATTTTCAC	
TcGNBP3-F	acgacgacgacaaggccatggATGAAAGAATTCTCCTTTGTAGTAGTGTT	recombinant expression of TcGNBP3
TcGNBP3-R	gtggtggtggtggtgctcgagTCAAAGGGCCCATATTTTCACA	
ds-*TcGNBP3*-F	TAATACGACTCACTATAGGGTATCAGTGAACAACAACGAGC	dsRNA synthesis of *TcGNBP3*
ds-*TcGNBP3*-R	TAATACGACTCACTATAGGGAGAACACACCCGCCGTACA	
ds-*GFP*-F	TAATACGACTCACTATAGGGTGGTCCCAATTCTCGTGGAAC	dsRNA synthesis of *GFP*
ds-*GFP*-R	TAATACGACTCACTATAGGGCTTGAAGTTGACCTTGATGCC	
*TcGNBP3*-qF	ACATCACAAAGGCTAAACACG	*TcGNBP3* transcriptional level
*TcGNBP3*-qR	GGAGACTTTTCTTTGTCTAGTA	
*Tcrps3*-qF	TCAAATTGATCGGAGGTTTG	internal control transcriptional level
*Tcrps3*-qR	GTCCCACGGCAACATAATCT	

### Bioinformatic analysis of TcGNBP3

*TcGNBP3* was characterized through integrated bioinformatic analyses [38]: BLASTX for homology search (*E*-value threshold of ≤1e^−22^, identity ≥31.94% and coverage ≥25%), MEGA 7.0 for phylogenetic tree reconstruction (neighbor-joining, *p*-distance, 1000 bootstrap replicates, and uniform rates and pairwise deletion with cutoff at 50%), SignalP 4.1 for signal peptide prediction, SMART for domain identification, ClustalW2 for multiple sequence alignment, and ExPASy for physicochemical property calculation.

### Quantitative real-time PCR (qRT-PCR) analyses of the expression profiles of TcGNBP3

Samples were collected from multiple individuals at distinct developmental stages: early eggs (EE, 1-day-old, ~50 mg), late eggs (LE, 3-day-old, ~50 mg), early larvae (EL, 1-day-old, ~50 mg), late larvae (LL, 20-day-old, *n* = 3), early pupae (EP, 1-day-old, *n* = 3), late pupae (LP, 5-day-old, *n* = 3), early adults (EA, 1-day-old, *n* = 3), and late adults (LA, 10-day-old, *n* = 3). For tissue-specific analysis, hemolymph, fat body, gut, epidermis, and central nervous system were dissected from 15-day-old (fifth-instar) larvae.

Larvae (*n* = 150) were injected with 200 nL of infection buffer (IB: 0.373 g/L KCl, 0.038 g/L Na_3_PO_3_·12 H_2_O) containing either *Escherichia coli* (~7.8 × 10^4^ cells) or *Staphylococcus aureus* (~7.0 × 10^4^ cells). The bacterial strains were cultured in Luria-Bertani medium (LB) at 37 °C overnight, harvested during the exponential phase by centrifugation at 6,000 g for 5 min at room temperature, and washed three times with IB. Bacterial concentration was estimated by measuring the optical density at 460 nm using a spectrophotometer. Control larvae (*n* = 80) received IB alone. Following injection, three surviving individuals per group were randomly sampled at 6, 12, 24, 36, 48, 60, and 72 h post-injection for expression analysis.

Total RNA extraction and cDNA synthesis were performed as described in the “Cloning and sequencing of *TcGNBP3*” section above. A pair of specific primers *TcGNBP3*-qF and *TcGNBP3*-qR (Table 1) were designed using the Primer program version 5.0. According to the manufacturer’s instructions, qRT-PCR was performed on the StepOnePlus real-time fluorescent quantitative PCR system (ABI, USA) to detect the expression pattern of *TcGNBP3*. The reaction volume of qPCR was 10 μL, including 0.5 μL of each specific primer, 1 μL of cDNA, 5 μL of SYBR Green Master Mix (Vazyme, Nanjing, China), and 3 μL of ddH_2_O. The expression level of *TcGNBP3* relative to ribosomal protein S3 (*Tcrps3*, ID: 659,167) (2^−ΔΔCT^) was calculated by comparing the threshold cycle method. Each experiment was repeated three times.

### Molecular docking of LPS, PGN, Glu and TcGNBP3

We conducted molecular docking experiments to predict whether TcGNBP3 can have a binding function with PAMPs. The three-dimensional structure of TcGNBP3 protein was preprocessed using Swiss-MODEL server (https://swissmodel.expasy.org/). AutoDock Vina 1.1.2 was utilized to perform molecular docking simulations between TcGNBP3 and carbohydrate ligands. We employed Discovery Studio 2019 for 3D structural analysis and docking visualization of TcGNBP3.

### Recombinant expression and purification of TcGNBP3

The cDNA fragment encoding TcGNBP3 was amplified with primers TcGNBP3-F and TcGNBP3-R (Table 1) and cloned into the pET-32a (+TrxA +6×His) vector using the ClonExpress® II One Step Cloning Kit (Vazyme, Nanjing, China). The resulting recombinant plasmid, pET-32a-TcGNBP3, was transformed into *E. coli* BL21 (DE3) for expression. The recombinant protein rTcGNBP3 was purified using a Sepharose™ 6 Fast Flow column (GE Healthcare, USA), resolved by 12.5% SDS-PAGE, and stained with Coomassie brilliant blue. For Western blot analysis, the protein was transferred to a PVDF membrane following electrophoresis. Protein concentration was measured using a quantitative detection kit (Jiancheng, Nanjing, China).

### Binding and agglutination of TcGNBP3 with microorganisms

The binding affinity of TcGNBP3 to polysaccharides (LPS, PGN, and Glu) was analyzed by Enzyme-linked immunosorbent assay (ELISA). Microtiter plates (96-well, Corning, USA) were coated with 80 µg/mL of each polysaccharide (50 µL/well), followed by incubation with serially diluted rTcGNBP3 (100 µL/well). Binding was detected using rabbit anti-His polyclonal antibody (Transgen, China) and HRP-conjugated goat anti-rabbit IgG (Transgen, China), with 3,3,’5,5’-tetramethylbenzidine (TMB) substrate (Beyotime, China) for color development. The reaction was stopped after 15 min with 2 M sulfuric acid, and absorbance was measured at 450 nm (Synergy H1M, BioTek, USA). Bovine serum albumin (BSA) served as the negative control, with triplicate measurements for each sample.

The microbial binding activity of TcGNBP3 was examined using five bacterial strains: three Gram-positive (*S. aureus*, *Bacillus subtilis* and *Bacillus thuringiensis*) and two Gram-negative (*E. coli* and *Pseudomonas aeruginosawere*). Bacteria were washed and suspended in sterile TBS (137 mM NaCl, 3 mM KCl, 25 mM Tris-HCl, pH 7.6) to ~2 × 10^8^ cells/mL, then incubated with rTcGNBP3 (containing 10 mM CaCl_2_) for 1 h at room temperature with gentle rotation. After five TBS washes, bound proteins were eluted with 7% sodium dodecyl sulfate (SDS) and analyzed by Western blot. BSA replaced rTcGNBP3 in negative controls, with triplicate experiments performed.

The same five bacterial strains were used to assess rTcGNBP3’s agglutination activity. In 10 mM Ca^2 +^, 25 μL bacterial suspension was combined with 25 μL rTcGNBP3 (200 μg/mL final concentration) or BSA (200 μg/mL, negative control), then incubated at room temperature for 1 h. Agglutination was examined under 1,000× magnification using bright-field microscopy. Three biological replicates were set up in each group to ensure the reliability of the results.

### dsRNA-mediated RNA interference experiment

The double-stranded RNA (dsRNA) targeting *TcGNBP3* or *GFP* (control) was synthesized using a T7 transcription kit (Takara, Dalian, China). Gene-specific primers (ds-*TcGNBP*3-F/R or ds-*GFP*-F/R, Table 1) were used to prepare DNA templates for *TcGNBP3*-dsRNA or *GFP*-dsRNA by PCR amplification. DNA templates were amplified with gene-specific primers followed by *in vitro* transcription. The dsRNA integrity was verified by agarose gel electrophoresis, and concentration was determined spectrophotometrically (NanoDrop 2000, USA).

In order to determine the efficiency of gene silencing, *T. castaneum* at 13 days of age was selected and randomly divided into two groups: the experimental group was injected with 200 ng of *TcGNBP3*-dsRNA in a volume of 200 nL, and the control group was injected with the same dose of *GFP*-dsRNA. Total RNA was isolated from three individuals in each group on days 1, 3 and 5 after treatment, and the transcription level of *TcGNBP3* gene was detected by qRT-PCR (method reference section 2.4) to verify the knockdown effect. At the 72 h after injecting ds-*TcGNBP3* into *T. castaneum*, *E. coli* was injected, and then the expression of related genes (including transcription factors (TFs), antimicrobial peptides (AMPs) and prophenoloxidase (proPO) activation system) and the proPO activity was detected at the 24th h after bacterial stimulation. Corresponding gene identifiers for *T. castaneum* TFs, AMPs and proPO are provided in Reference [39]. Then, the hemolymph of the larvae in the control group (WT + *E. coli*, ds-*GFP* + *E. coli*) and the experimental group (ds-*TcGNBP3* + *E. coli*) was separately extracted and then was added with pre-cooled buffer (0.001% Tween-20, 1 mM CaCl2, 20 mM Tris-HCl, pH 7.5). PO activity was determined using dopamine as a substrate on a 96-well microplate reader, and the absorbance was read at 490 nm, with one unit of activity representing an increase of 0.001 absorbance units per minute. The semi-quantitative analysis of melanin deposition gray values using ImageJ. The above experiments were set up three independent biological replicates.

### Survival experiment

To assess RNAi’s impact on beetle immunity, 80 healthy 13-day-old larvae were equally divided into four groups: *TcGNBP3*-dsRNA and *GFP*-dsRNA controls, each challenged with either *E. coli* (7.8 × 10^4^ cells) or *S. aureus* (7.0 × 10^4^ cells) via 200 nL injections. Mortality was recorded every 12 h, with survival curves generated using Kaplan-Meier analysis in GraphPad Prism 5 (GraphPad Software, USA) and group differences analyzed by Log-rank test.

### Statistical analyses

Statistical analyses were performed using SPSS 13.0 (SPSS Inc., Chicago, IL, USA). Gene expression data from multiple groups were compared by one-way ANOVA followed by Tukey’s honestly significant difference (HSD) post-hoc test, with significant differences indicated by assigned shared lowercase letters (*p* < 0.05). Comparisons between two groups were conducted using Student’s t-test, with significance denoted as *p < 0.05, **p < 0.01, and ***p < 0.001. All data are presented as the mean ± SD from three independent experiments.

## Results

### Molecular characteristics and bioinformatics analysis of TcGNBP3

The *TcGNBP3* genecontains a 1,446-bp open reading frame (ORF) that translates into a 481-amino acid protein (predicted 55 kDa, pI 5.76). Bioinformatic analysis revealed an N-terminal signal peptide (M1-A18) in the TcGNBP3 protein sequence. Domain architecture analysis identified two functional domains: a CBM39 located at the N-terminal region (E21-P133, 113 aa) and a GH16 domain positioned at the C-terminus (D234-A414, 181 aa) (Figure S1). Comparative sequence analysis revealed substantial structural divergence among GNBP family proteins, TcGNBP1(ID: 658,535), TcGNBP2 (ID: 660,349) and TcGNBP3 (ID: 660,764), with significant differences in both domain organization and amino acid sequence homology (Figure S2).

Phylogenetic reconstruction demonstrated that TcGNBP3 clusters most closely with orthologs from *Tribolium madens* and *Tenebrio molitor*, while TcGNBP1, TcGNBP2, and TcGNBP3 form three distinct clades within the evolutionary tree (Figure. S3). The deep phylogenetic separation of these paralogous genes suggests that their divergence predated the emergence of the respective insect orders.

### Spatiotemporal expression of TcGNBP3

**Figure 1.**
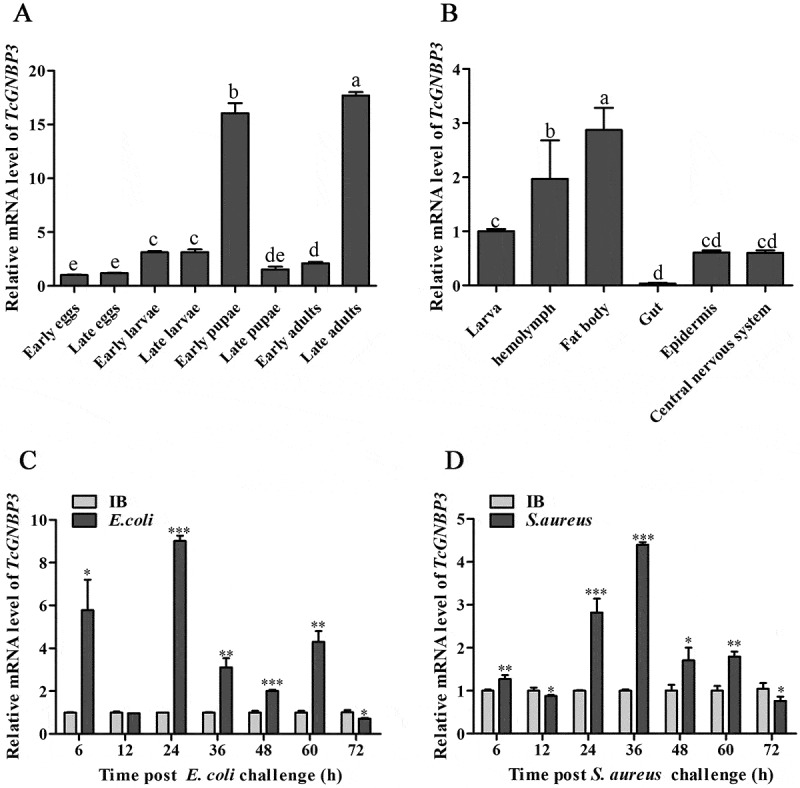
Spatiotemporal expression of *TcGNBP3*. (A) Expression profile of *TcGNBP3* during different developmental stages of the insect, relative to the early egg stage. (B) Tissue-specific expression in 15-day-old larvae. Expression in each tissue is presented relative to a calibrator sample consisting of total RNA from a whole larva (“whole-body sample”). (C, D) Temporal expression after challenge with *E. coli* (C) or *S. aureus* (D) versus IB-injected controls. All qRT-PCR data were normalized to the housekeeping gene *Tcrps3*. Significant differences are indicated by different letters (one-way anova with Tukey’s test, *p* < 0.05) for multiple groups in (A, B), or by asterisks (Student’s t-test, *p < 0.05, **p < 0.01, ***p < 0.001) for comparisons between two groups at each time point in (C, D). Data are mean ± SD (*n* = 3).

To investigate the functional role of *TcGNBP3*, we employed qRT-PCR to analyze its expression patterns across various developmental stages, tissues, and following bacterial challenge. Results showed that *TcGNBP3* was expressed at all developmental stages. Among them, the relative mRNA transcription level was highly expressed in EP and LA stages (Figure 1(A)). The tissue expression profile showed that the highest expression was in fat body, followed by hemolymph (Figure 1(B)). In order to preliminarily study the possible role of *TcGNBP3* in innate immunity, bacteria were injected into 15-day-old larvae. Compared with the control group, the abundance of *TcGNBP3* was significantly up-regulated at 6, 24, 36, 48 and 60 h after infection with *E. coli* and *S. aureus*, respectively (Figure 1(C,D)). These results suggest that *TcGNBP3* may be involved in the innate immune response of *T. castaneum*.

### Molecular docking of PAMPs and TcGNBP3

**Figure 2.**
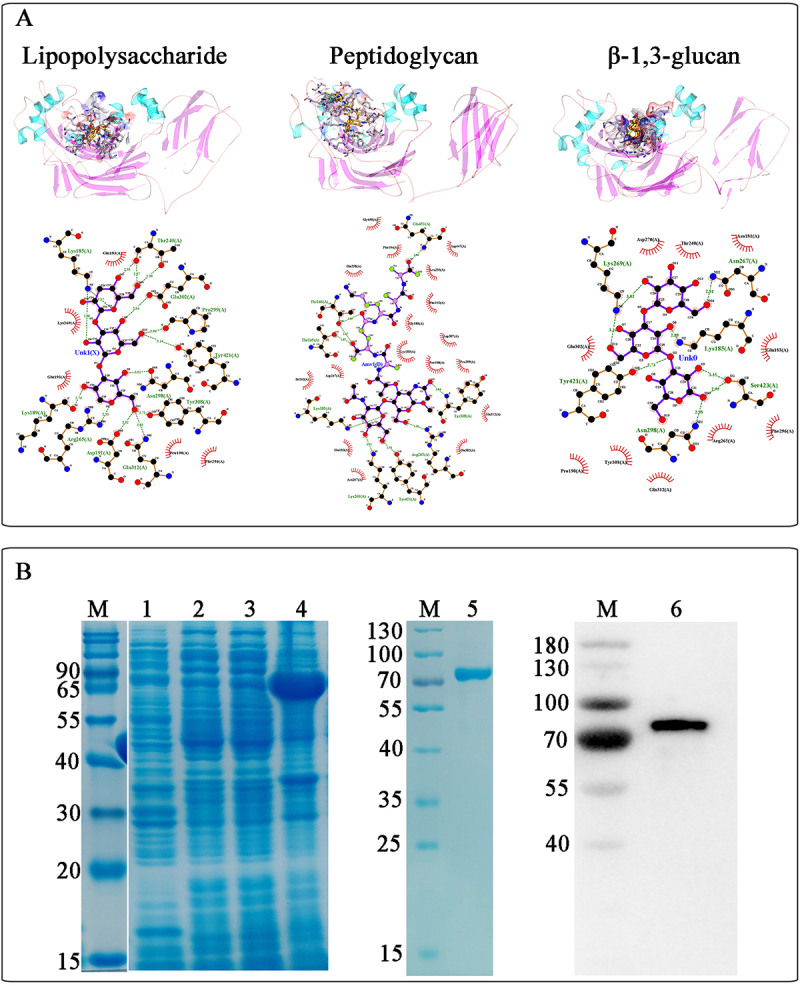
Molecular docking visualization of TcGNBP3 with carbohydrate ligands and analysis of recombinant protein expression. (A) The complexes of TcGNBP3 with core oligosaccharide of LPS, glucosamyl muramyl pentapeptide and β-1, 3-glucan.(B) SDS-PAGE and western blotting analysis of the recombinant TcGNBP3. Lane M: the protein molecular weight marker; lane 1: the supernatant protein of *E. coli* with pET-32a; lane 2: precipitated protein from *E. coli* with pET-32a; lane 3: the supernatant protein of *E. coli* with pET-32a-TcGNBP3; lane 4: the precipitation protein of *E. coli* with pET-32a-TcGNBP3; lane 5: purified recombinant TcGNBP3 protein; lane 6: western blot based on the sample of lane 5.

TcGNBP3’s N-terminal domain contains a characteristic hydrophobic ligand-binding pocket (Figure 2(A)) that accommodates three small molecules. The pocket’s polar residues (glutamine, asparagine, arginine, and lysine) mediate electrostatic interactions with polysaccharide oxygen atoms (hydroxyl, epoxide, and aldehyde groups). Tyrosine and threonine stabilize the binding of polysaccharide molecules through hydrogen bonding. Moreover, proline, isoleucine, phenylalanine, and glycine in the active pocket provide a stable hydrophobic environment for polysaccharide binding.

### Expression and purification of recombinant TcGNBP3 in vitro

The recombinant plasmid pET-32a-*TcGNBP3* was expressed in *E. coli* BL21 (DE3), yielding a 70 kDa protein band (Figure 2(B), lane 4) upon SDS-PAGE analysis, matching the predicted molecular weight. Subsequent purification using Ni Sepharose™ 6 Fast Flow column was confirmed by both SDS-PAGE (Figure 2(B), lane 5) and Western blot analysis (Figure 2(B), lane 6), demonstrating successful isolation of rTcGNBP3.

### Microbial binding and aggregation of TcGNBP3

**Figure 3.**
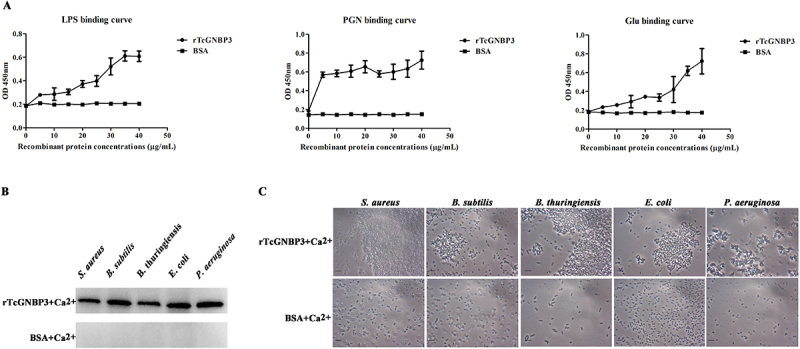
Binding and agglutination activities of rTcGNBP3 protein. (A) ELISA analysis of rTcGNBP3 binding to LPS, PGN, and Glu. (B) Western blot detection of purified rTcGNBP3 binding to Gram-positive (*S. aureus*, *B. subtilis*, *B. thuringiensis*) and Gram-negative bacteria (*E. coli*, *P. aeruginosa*). (C) Calcium-dependent microbial agglutination activity of rTcgnbp3 against the same bacterial strains. BSA served as a negative control. Agglutination was observed under a compound light microscope (1,000 × magnification; scale bar = 10 μm).

GNBPs, as PRRs, mediate pathogen recognition and subsequent immune activation. To investigate the possible interactions between TcGNBP3 and microorganisms, firstly, ELISA was used to detect whether TcGNBP3 could bind to polysaccharides (LPS, PGN and Glu). ELISA analysis revealed dose-dependent binding of rTcGNBP3 to LPS, PGN, and Glu (Figure 3(A)). In addition, at the same concentration, TcGNBP3 has higher binding activity to PGN. Binding assays demonstrated specific interactions between rTcGNBP3 and both Gram-positive (*S. aureus*, *B. subtilis* and *B. thuringiensis*) and Gram-negative bacteria (*E. coli* and *P. aeruginosawere*) (Figure 3(B)), with no binding observed for the BSA control. To determine whether the binding activity of rTcGNBP3 protein can induce microbial agglutination, bacterial agglutination experiments were performed, and it was found that rTcGNBP3 had agglutination activity for all detected bacteria (Figure 3(C)).

### RNA interference of TcGNBP3 and its effect in T. castaneum

**Figure 4.**
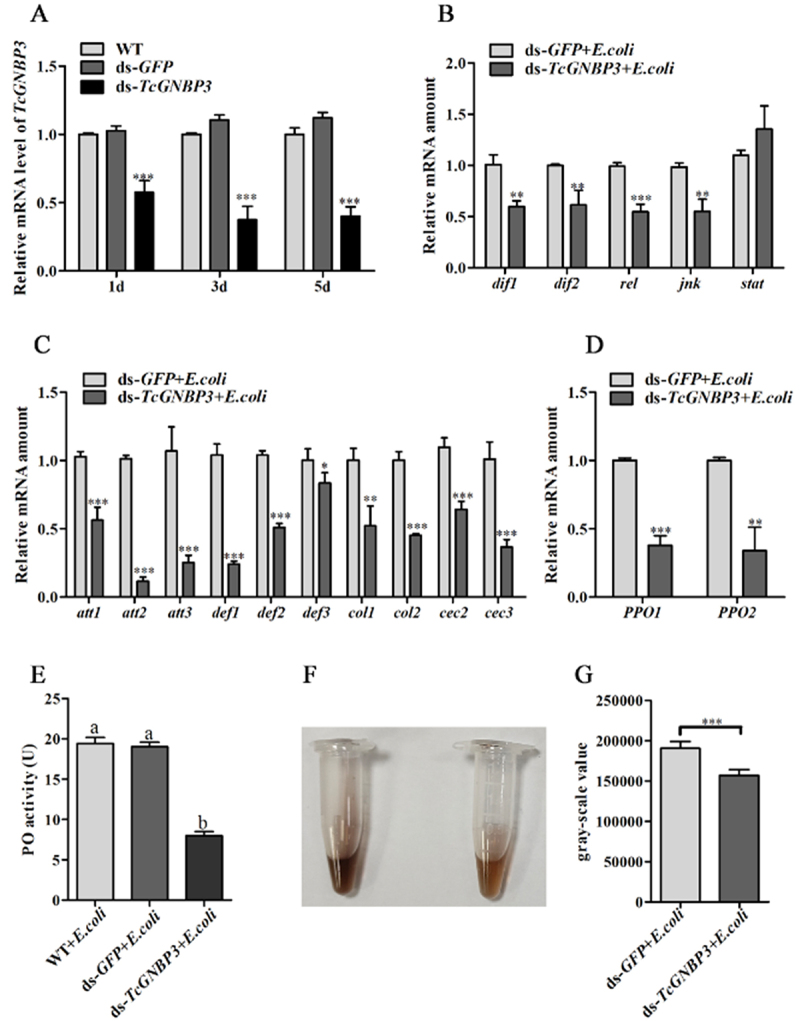
Immune response disruption upon *TcGNBP3* knockdown in *T. castaneum* under *E. coli* challenge. (A) RNAi efficiency of *TcGNBP3*. Expression levels of transcription factors (B), AMPs (C) and *proPO* (*PPO1* and *PPO2*) (D) After *E. coli* challenge in *TcGNBP3*-silenced larvae. (E) PO activity assay after *TcGNBP3* silencing under *E. coli* challenge. (F) Photograph observed an obvious reduction in the melanization of *TcGNBP3* after RNAi treatment, compared to the control group (ds-*GFP* + *E. coli*). (G) Semi-quantitative analysis of melanin deposition gray values from (F) Using ImageJ. Data are presented as mean ± SD from three biological replicates. Significant differences are indicated by asterisks (Student’s t-test, *p < 0.05, **p < 0.01, ***p < 0.001) for comparisons between two groups (A, B, C, D, G), or by different letters (one-way anova with Tukey’s test, *p* < 0.05) for multiple groups in (E).

Timing expression analysis was performed by qRT-PCR to evaluate the efficiency of RNA interference-mediated gene knockdown for *TcGNBP3*. RNAi treatment significantly reduced larval *TcGNBP3* mRNA expression compared to *GFP*-dsRNA controls. The *TcGNBP3* gene expression level were 57.41 ± 8.83%, 37.49 ± 9.78% and 39.90 ± 7.09%, respectively, at 1, 3 and 5 days after intervention (Figure 4(A)). The results showed that the silencing of *TcGNBP3* could be effectively maintained at the transcriptional level during 1–5 days after injection, and the samples collected during this period were suitable for subsequent functional analysis.

We explored the potential role of *TcGNBP3* in *T. castaneum* by attacking *E. coli* after *TcGNBP3* RNAi treatment. RNAi-mediated knockdown of *TcGNBP3* significantly downregulated expression of immune-related TFs (*dif1, dif2, rel* and *jnk*) and AMPs (*att1-3, def1-3, cole1-2, cecr2-3*) (Figure 4(B,C)). These findings indicate that *TcGNBP3* coordinates multiple immune pathways and AMP regulation to bolster *T. castaneum*’s antimicrobial defense. Further studies demonstrated that silencing of *TcGNBP3* significantly downregulated the transcription levels of proPO (*PPO1* and *PPO2*) (Figure 4(D)) and reduced PO activity (Figure 4(E)). Moreover, quantitative grayscale analysis using ImageJ confirmed a marked decrease in melanin deposition in the *TcGNBP3*-dsRNA group compared to the control groups (Figure 4(F,G)). These results together suggest that *TcGNBP3* plays an important regulatory role in the activation of the PO system in *T. castaneum*.

### TcGNBP3 is involved in the immune-resistant survival of T. castaneum against bacteria

**Figure 5.**
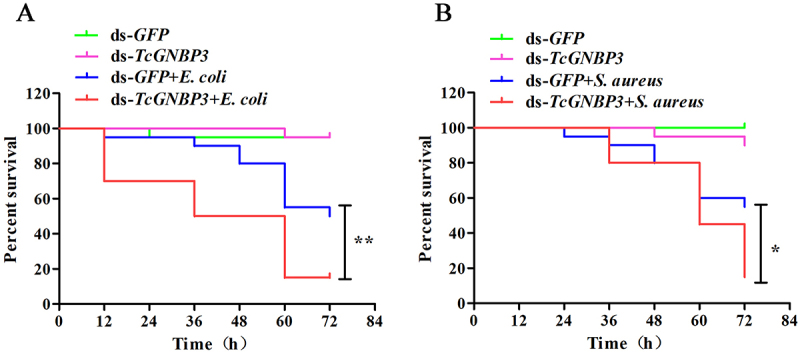
The survival rate of *TcGNBP3*-RNAi *T. castaneum* infected with *E. coli* (A) and *S. aureus* (B). The survival number of beetle was recorded every 12 h’ post-challenge. The experiments were repeated three times. *, significantly different from ds-*GFP* + *E. coli* or *S. aureus* controls by Gehan-Breslow-Wilcoxon test.

In order to confirm the relationship between *TcGNBP3* in *T. castaneum* and immune response, we studied the role of *TcGNBP3* in resistance to bacterial infection after RNAi. Kaplan–Meier survival curve analysis showed that the survival rate of larvae infected with *E. coli* (Figure 5(A)) or *S. aureus* (Figure 5(B)). *TcGNBP3* knockdown group was significantly lower than that of the control group. It indicates that *TcGNBP3* is a key effector molecule that regulates the antibacterial immune response of beetle.

## Discussion

As a well-established model organism in pest control research, *T. castaneum* provides critical insights into RNAi-based management strategies. Understanding its innate immunity mechanisms, particularly pathogen recognition proteins like GNBPs, is essential for developing targeted biocontrol approaches. Previous studies have demonstrated that GNBPs serve as key PRRs initiating immune responses against diverse pathogens [12,40], and their RNAi-mediated knockdown increases host susceptibility to microbial infections [18]. Despite recognizing GNBPs’ potential as RNAi targets, the functional characterization of these proteins in *T. castaneum* remains incomplete, particularly regarding their specific immune roles and regulatory mechanisms. Our study provides the first systematic analysis of TcGNBP3 (a GNBP from *T. castaneum*), elucidating its immune functions and assessing the impact of its RNAi-mediated knockdown on survival rates.

To elucidate the critical functions of *TcGNBP3* in insect innate immunity, we first analyzed its spatiotemporal expression patterns. Our study reveals that *TcGNBP3* was highly expressed during two critical developmental windows, the EP and LA stages. These periods represent particularly vulnerable phases in the insect life cycle: the EP stage involves extensive tissue reorganization during metamorphosis when the protective cuticle is compromised, while the LA stage coincides with increased environmental exposure during active foraging and reproduction [41,42]. The observed high expression of *TcGNBP3* during these immunologically challenging periods strongly suggests its essential role in providing continuous immune surveillance and defense. Further research has revealed that *TcGNBP3* exhibits elevated transcriptional levels in both the fat body and hemolymph. Consistent with known mechanisms of insect immunity, *GNBPs* are typically synthesized in the fat body and subsequently secreted into the hemolymph to mediate immune responses [43]. Our findings demonstrate significant *TcGNBP3* expression in these two crucial immune tissues [44], strongly suggesting its functional role in immune defense. A hallmark feature of the immune system is the pathogen-inducible expression of immune effector molecules following microbial challenge [45]. Our results demonstrated significant upregulation of *TcGNBP3* expression from 6 to 60 h post-infection with either *E. coli* or *S. aureus*. This temporal induction pattern mirrors the characteristic response of *GNBPs* to bacterial challenge observed across insect species. Notably, similar induction profiles have been reported for *GNBP* homologs in other insects: *A. pisum GNBP1*/*GNBP2* following microbial exposure [18,46] and *Helicoverpa armigera βGRP4* after fungal infection [47]. These conserved response patterns strongly support *TcGNBP3*’s crucial function in antimicrobial defense mechanisms in *T. castaneum*.

Comparative domain architecture analysis revealed significant structural divergence among *T. castaneum* GNBP family members. TcGNBP3 maintains complete functional domains, including an N-terminal CBM39 (E21-P133, 113 aa) and a C-terminal GH16 (D234-A414, 181 aa), while TcGNBP1 [38] and TcGNBP2 [48] have undergone evolutionary domain loss, retaining only a single functional domain each. This structural distinction correlates with sequence-level divergence, as evidenced by substantial variations in domain organization and amino acid homology among these paralogs. Phylogenetically, TcGNBP3 clusters closely with orthologs from related tenebrionidaes (*T. madens* and *T. molitor*), whereas TcGNBP1 and TcGNBP2 form independent clades, suggesting distinct evolutionary trajectories. Functional implications of these structural differences are noteworthy. Previous studies demonstrated that TcGNBP1 and TcGNBP2 perform pathogen recognition and immune activation through their single retained domains (GH16 and CBM39, respectively) [33,42]. The preservation of both ancestral domains in TcGNBP3 suggests either: functional differentiation from its paralogs, possibly involving more complex ligand interactions, or enhanced immune capacity through synergistic domain cooperation. This dual-domain architecture (N-terminal CBM39 and C-terminal GH16) may enable TcGNBP3 to recognize a broader spectrum of pathogens or/and initiate stronger immune responses compared to its single-domain counterparts.

The specific interaction between GNBPs and PAMPs serves as the critical first step for microbial pathogen recognition in insect innate immunity [49]. Molecular docking analysis identified specific PAMP-binding sites in TcGNBP3 for LPS, PGN, and β-1,3-glucan. ELISA validation confirmed dose-dependent binding to these microbial surface polysaccharides, which are characteristic components of bacteria and fungi [50]. These finding suggestion that TcGNBP3 exhibits broad-spectrum recognition and binding capability toward diverse pathogenic microorganisms. Subsequent binding experiments demonstrated that the rTcGNBP3 protein could bind to all five tested bacterial species, including three Gram-positive (*S. aureus*, *B. subtilis* and *B. thuringiensis*) and two Gram-negative bacteria (*E. coli*, *P. aeruginosa*). While some GNBPs/βGRPs exhibit specificity for particular bacterial species [51], most members of this family in insects, including TcGNBP3, display broad pathogen recognition capabilities, highlighting their crucial role in insect immune defense systems. For example, in *P. xylostella*, PxβGBP displays cross-kingdom binding properties against both bacterial (*S. aureus* and *E. coli*) and fungal (*Isaria cicadae*) pathogens [52]. Correspondingly, PGRP-S (*O. furnacalis*) and MBP (*M. sexta*) demonstrate broad-spectrum recognition of *S. aureus* and *E. coli* [53,54]. In summary, these findings demonstrate that TcGNBP3 serves as a crucial pattern recognition molecule in microbial identification through its broad polysaccharide-binding specificity.

As a key innate immune mechanism in insects [55], GNBPs/βGRPs-mediated microbial agglutination exhibits broad-spectrum activity against diverse pathogens, including fungi, bacteria, parasites, and viruses. *Candida albicans* exhibited marked cellular aggregation in the presence of recombinant *Drosophila* GNBP3 but remained dispersed when treated with BSA [15]. *Sogatella furcifera* βGRP1 and βGRP2 from exhibit strong binding and agglutination activity against *E. coli* and *S. aureus* [56]. Similarly, a βGRP in *Anatolica polita* recognizes and aggregates *Saccharomyces cerevisiae*, *S. aureus*, and *E. coli* [57]. Our experimental data demonstrate that rTcGNBP3 induces agglutination in all tested bacterial strains, revealing its broad-spectrum microbial aggregation capability. Collectively, these findings suggest that TcGNBP3 acts as a PRR, contributing to immune defense through broad-spectrum PAMP recognition and bacterial agglutination.

Upon recognition of PAMPs, insect GNBPs/βGRPs primarily enhance innate immunity through activation of the Toll/IMD signaling pathways and/or the proPO system [58,59]. It is well known that the Toll and IMD signaling pathways can regulate the expression of AMPs [12,60]. GNBP3 triggers the Toll signaling pathway, resulting in transcriptional activation of the *Drosomycin* (*drs*) AMP gene in *Drosophila* [61]. Interestingly, the *Drosophila* immune system exhibits pathway-specific responses: Toll pathway predominantly responds to Gram^+^ bacterial and fungal infections, mainly controlling *drs* expression., while the IMD pathway responds mainly to Gram^−^ bacteria and controls AMPs such as *Diptericin* [62,63]. Unlike this, *T. castaneum* usually activates both the Toll and IMD signaling pathways simultaneously when it is infected by the same microorganism [64]. The results show that *TcGNBP3* knockdown significantly downregulated 10 AMP genes (*att1*, *att2*, *att3*, *def1*, *def2, def3*, *cole1*, *cole2*, *cecr2* and *cecr3*) and four immune-related transcription factors (*dif1*, *dif2*, *rel*, and *jnk*) in *E. coli*-challenged *T. castaneum*. Current understanding establishes that the Toll signaling pathway primarily regulates dorsal-related immunity factors (*dif1* and *dif2*), while the IMD pathway predominantly activates transcription factors relish (*rel*) and JNK kinase [45,65]. These findings suggest that *TcGNBP3* modulates AMP expression in *T. castaneum* through dual regulation of both IMD and Toll signaling pathways during *E. coli* infection. Beyond their established role in Toll/IMD pathway activation, some GNBPs additionally participate in proPO system activation. For example, the specific recognition of β-1,3-glucan by *M. sexta* βGRP2 induces proteolytic activation of hemolymph proHP14, leading to subsequent initiation of the proPO cascade [66]. RNAi-mediated knockdown of *ApGNBPs* in *A. pisum* significantly attenuated PO activity in the hemolymph, confirming their functional involvement in this enzymatic cascade [18,46]. In the study, RNAi-mediated silencing of *TcGNBP3* reduced *PPO1*/*PPO2* expression, diminished hemolymph PO activity, and impaired melanization capacity following *E. coli* challenge in *T. castaneum*. These findings collectively demonstrate that *TcGNBP3* plays a pivotal role in regulating both the Toll/IMD signaling pathways and proPO system activation during immune responses.

Emerging studies have demonstrated the potential of RNAi technology as an effective component in integrated pest management systems [67,68], showing a better effect when combined with biocontrol bacteria. Application of RNAi to knockdown *NlGRP3* expression increases the effectiveness of *Metarhizium anisopliae* as a biological control agent for brown planthopper *Nilaparvata lugens* management [69]. RNAi-mediated suppression of *GNBP2* via dsRNA ingestion enhanced *Reticulitermes termite* susceptibility to naturally occurring *M. anisopliae*, resulting in elevated mortality [30]. Similarly, the silencing of immune genes *Rel1* and *Rel2* in *Aedes aegypti* mosquitoes significantly compromised their physiological fitness and enhanced susceptibility to entomopathogenic fungal infections [70]. Given the crucial role of *TcGNBP3* in *T. castaneum* innate immunity, we investigated its specific contribution to resistance against *E. coli* and *S. aureus*. Knockdown of *TcGNBP3* by dsRNA significantly compromised the beetle’s antibacterial defense against both pathogens. These findings substantiate the practical application and enhanced efficacy of combining RNA interference with pathogen approaches for *T. castaneum* control. As a mode insect, *T. castaneum* has been widely employed in identifying novel coleopteran pest control targets [32]. Therefore, our findings demonstrate that *TcGNBP3* represents a promising molecular target for coleopteran pest management

In summary, our study establishes TcGNBP3 as a versatile pattern recognition receptor that detects a broad spectrum of PAMPs and binds various pathogenic microbes. Functional impairment of TcGNBP3 via RNAi compromised immune activation, rendering hosts more susceptible to bacterial infection and highlighting its essential role in antibacterial immunity. These findings not only advance the understanding of insect innate immunity but also point to TcGNBP3 as a promising target for RNAi-based pest control and microbe-induced management strategies.

## Data Availability

The data that support the findings of this study are openly available in EU Open Research Repository Zenodo at https://doi.org/10.5281/zenodo.16711359 [71].
